# Dynamic egg color mimicry

**DOI:** 10.1002/ece3.2187

**Published:** 2016-05-24

**Authors:** Daniel Hanley, Michal Šulc, Patricia L. R. Brennan, Mark E. Hauber, Tomáš Grim, Marcel Honza

**Affiliations:** ^1^ Department of Zoology and Laboratory of OrnithologyPalacký University17. listopadu 50Olomouc771 46Czech Republic; ^2^ Institute of Vertebrate BiologyAcademy of Sciences of the Czech Republicv. v. i., Květná 8603 65BrnoCzech Republic; ^3^ Department of EcologyFaculty of ScienceCharles University in PragueViničná 7128 44Prague 2Czech Republic; ^4^ Department of Biological SciencesMount Holyoke CollegeSouth HadleyMassachusetts01074USA; ^5^ Organismic & Evolutionary Biology Graduate ProgramUniversity of Massachusetts, AmherstAmherstMassachusetts01003USA; ^6^ Department of PsychologyHunter College and the Graduate CenterThe City University of New York695 Park AvenueNew York CityNew York10065USA

**Keywords:** Avian vision, brood parasitism, coevolution, common cuckoo, mimicry, pigments

## Abstract

Evolutionary hypotheses regarding the function of eggshell phenotypes, from solar protection through mimicry, have implicitly assumed that eggshell appearance remains static throughout the laying and incubation periods. However, recent research demonstrates that egg coloration changes over relatively short, biologically relevant timescales. Here, we provide the first evidence that such changes impact brood parasite–host eggshell color mimicry during the incubation stage. First, we use long‐term data to establish how rapidly the *Acrocephalus arundinaceus* Linnaeus (great reed warbler) responded to natural parasitic eggs laid by the *Cuculus canorus* Linnaeus (common cuckoo). Most hosts rejected parasitic eggs just prior to clutch completion, but the host response period extended well into incubation (~10 days after clutch completion). Using reflectance spectrometry and visual modeling, we demonstrate that eggshell coloration in the great reed warbler and its brood parasite, the common cuckoo, changes rapidly, and the extent of eggshell color mimicry shifts dynamically over the host response period. Specifically, 4 days after being laid, the host should notice achromatic color changes to both cuckoo and warbler eggs, while chromatic color changes would be noticeable after 8 days. Furthermore, we demonstrate that the perceived match between host and cuckoo eggshell color worsened over the incubation period. These findings have important implications for parasite–host coevolution dynamics, because host egg discrimination may be aided by disparate temporal color changes in host and parasite eggs.

## Introduction

Birds’ eggs display a range of patterns and colors that vary from blue‐green to brown (Hanley et al. 2015a). Many factors are known to affect expression of avian eggshell color including genetics, health status and age of the laying female, diet, yearly fluctuations of rainfall and temperature, and pollution (reviewed in Cherry and Gosler 2010). Avian eggshell coloration has important fitness consequences. For example, cryptic and disruptive coloration can increase clutch survival (Kilner 2006; Stoddard et al. 2011), and some eggshell colors may protect the embryo against harmful ultraviolet radiation (Lahti and Ardia 2016), synchronize the circadian rhythms of the developing embryo, or even serve as a postmating signal of female quality to males (Cherry and Gosler 2010). In addition, coevolutionary arms races between hosts and brood parasites can result in adaptations for egg discrimination on part of hosts, and in turn, improved eggshell mimicry on part of their parasites (Dawkins and Krebs 1979; Stoddard and Stevens 2010). All of these functions could be impacted if eggshell coloration changes over time.

Variation in avian eggshell coloration is controlled mainly by two pigments, protoporphyrin and biliverdin, that are embedded within the eggshell's calcium carbonate matrix (Kennedy and Vevers 1976; Hanley et al. 2015a), and is also influenced by eggshell structure and the proteinaceous cuticle layer found on many birds’ eggs (Fecheyr‐Lippens et al. 2015; Igic et al. 2015a). Eggshell coloration is known to change over decades (Cassey et al. 2010; Hanley et al. 2013a), years (Cassey et al. 2012), weeks (Moreno et al. 2011), and even days (Hanley et al. 2013b; Navarro and Lahti 2014). As eggshell colors can change in a matter of days, the timing of the spectral measurements (i.e., age of the egg at the time of measurement) may represent an important confounding factor. Nonetheless, the majority of published studies do not report the precise timing during the laying cycle when eggshell coloration was assessed (e.g., Hanley and Doucet 2009). In other cases, measurement times varied widely, from laying until hatching (Honza et al. 2012). Short‐term changes (i.e., within the incubation period) in coloration and brightness are relatively small (averaging from 2% to 7% reflectance), but statistically significant (Moreno et al. 2011; Navarro and Lahti 2014). However, it remains unclear whether these changes in color are visually detectable by birds (sensu Dearborn et al. 2012), which is critical for the adaptive significance of avian eggshell coloration shifts. Within the context of host–brood parasite coevolution, even relatively small perceivable differences in eggshell coloration can result in substantial increases in host rejection rates (Honza et al. 2011; Hauber et al. 2015).

**Figure 1:**
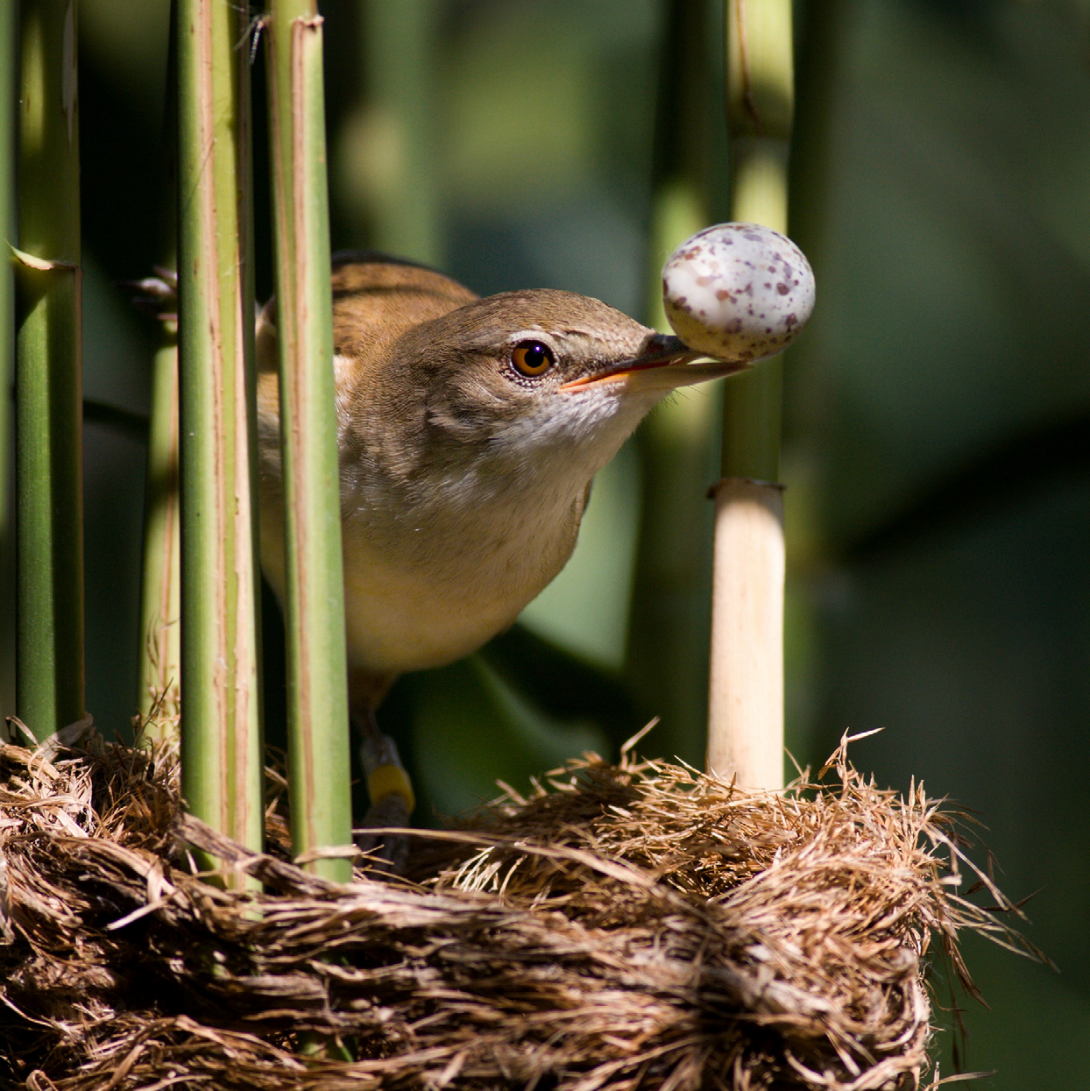
The great reed warbler *Acrocephalus arundinaceus* is a frequently parasitized host of the common cuckoo *Cuculus canorus* across Europe. To remove foreign eggs from its nest, this species pierces the egg with its bill, a practice known as puncture rejection. Here, we illustrate a great reed warbler removing an egg from its nest. Photograph credit: Oldřich Mikulica.

**Figure 2:**
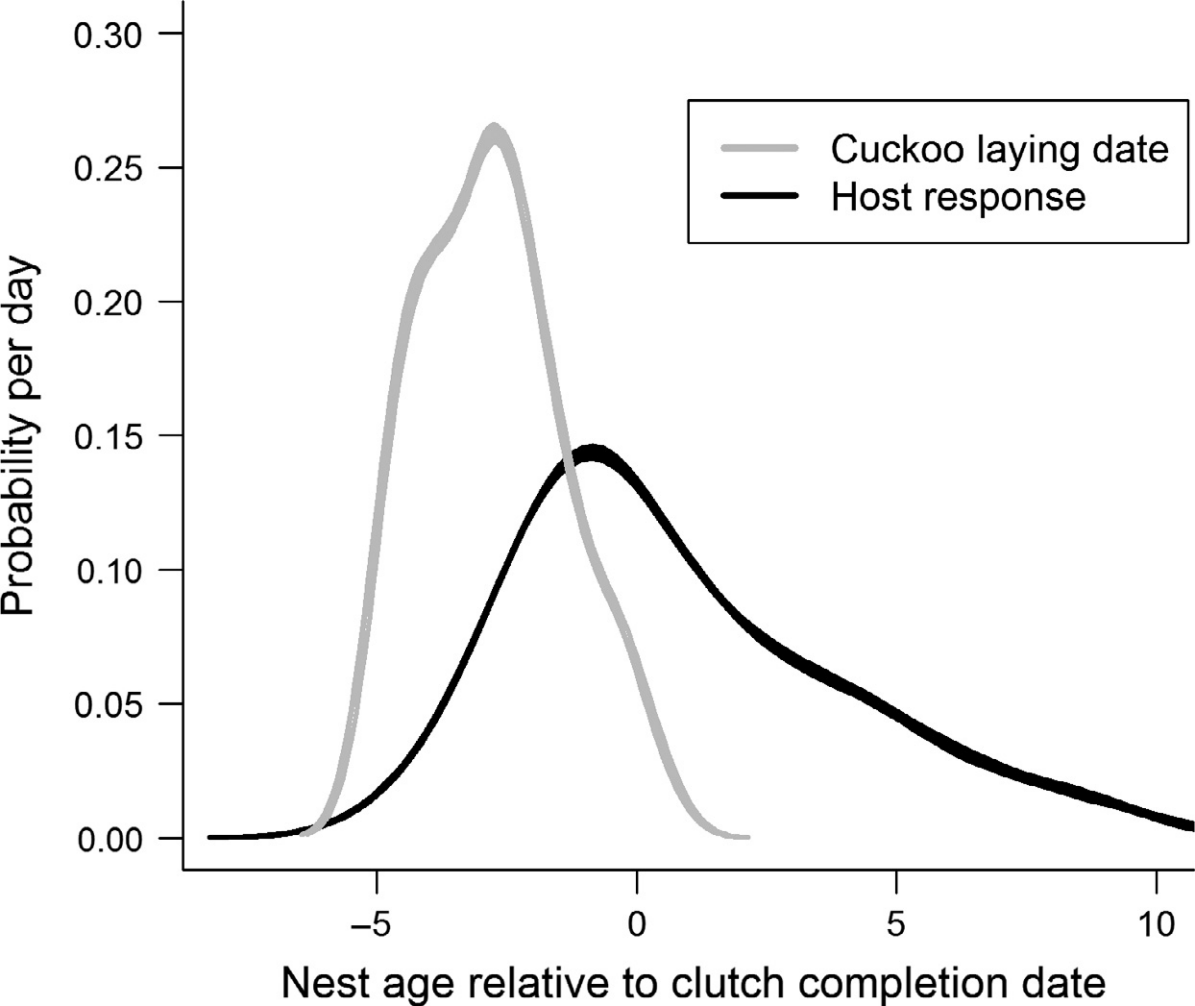
Probability density functions describing great reed warbler rejection (*N* = 91) of a naturally laid common cuckoo egg (black lines) and laying dates of parasitic cuckoo eggs (light gray), relative to clutch completion (set to zero). Uncertainty of event dates is accounted for via bootstrap, where thicker and thinner portions of the lines indicate areas of relatively less or more certainty (for further details, see “Material and Methods”).

Here, we investigated whether changes in eggshell coloration occur on a timescale relevant for egg rejection behavior, and on a perceptual scale that is visually detectable to an avian receiver, and whether these changes influence eggshell mimicry. First, we used long‐term monitoring data, to establish how rapidly host *Acrocephalus arundinaceus* Linnaeus (great reed warbler, hereafter warbler) responded to naturally laid parasitic *Cuculus canorus* Linnaeus (common cuckoo, hereafter cuckoo) eggs (Fig. 1). Previous research on this host has shown that perceived eggshell coloration is an important cue for warblers’ egg rejection (Moskát et al. 2014b; Hauber et al. 2015), while in our study population age, host genotype, and ambient light do not affect host egg rejection (Honza et al. 2011; Procházka et al. 2014). Second, we measured warbler and parasitic cuckoo eggs at three time points spanning this empirically derived host response period (Fig. 2), and quantified whether eggshell reflectance spectra changed over time, and how those changes were distributed across the avian visual spectrum. Then, using receptor noise‐limited visual models (Vorobyev and Osorio 1998; Vorobyev et al. 1998), we evaluated whether the magnitude of perceivable color changes was similar between the host and its parasite. Previous studies have shown that hosts and parasites in our study population contain similar eggshell pigment chemistry (Igic et al. 2012), and eggshell color changes have been attributed to the pigment degradation (Moreno et al. 2011). Therefore, we predicted that (1) both host and parasite eggshell colors changed similarly across the incubation period, and thus, (2) mimicry should remain constant. However, the potential for eggshell color changes to influence mimicry is a completely unexplored possibility, and dynamic color changes may provide an important advance in our understanding of natural colors and coevolution.

## Material and Methods

### Study area and field measurements

The study was conducted on a color‐ringed warbler population at a fish pond area near Hodonín (48°51′N, 17°07′E), Czech Republic, between 2008 and 2015 from May to June. We systematically searched for warbler nests in the littoral vegetation surrounding the ponds. Overall, we found 825 great reed warbler nests, 91 of which were found naturally parasitized by the cuckoo during the egg stage for which latency to host response could be estimated. No eggs were experimentally removed or added to any of these nests. Losses of two host eggs and appearance of one cuckoo egg were attributed to cuckoo egg removal (Moksnes et al. 2000), not rejection errors (i.e., the host removing its own egg without harming the parasitic egg, Stokke et al. 2002) on part of the host. We consider responses as either acceptance or rejection of the natural cuckoo eggs through either desertion or ejection. Desertion was regarded as a type of host rejection because we found that parasitized nests were deserted substantially more often than nonparasitized nests (78 of 299 parasitized nests and 12 of 179 nonparasitized nests, respectively, were deserted; *χ* ^2^ = 26.27, df = 1, *P *<* *0.0001). For this calculation, we only used nests that survived until hatching. Moreover, we used only nonparasitized nests for which we knew complete egg‐laying sequence in order to minimize the possibility that they were imperceptibly parasitized. Desertion as a response was also shown in other great reed warbler populations (Moskát and Honza 2002; Moskát et al. 2008). In contrast with research based on responses to experimental parasitism (Hanley et al. 2015b; Igic et al. 2015b; Šulc et al. 2016), typically using a 3‐ to 6‐day cutoff period (Spottiswoode 2013; Hanley et al. 2015b), we imposed no artificial cutoff (similar to, Davies and Brooke 1989), because we assume that responding to natural parasitism events poses a substantial cognitive challenge for hosts and because natural selection imposes no cutoff.

We investigated how long cuckoo eggs remained in the nest before hosts responded (hereafter, latency to response). We were able to estimate the latency to response (in days) in 91 parasitized nests (58 ejections of cuckoo egg and 33 desertions of the nest). However, because nests were not checked daily, there was variation in the accuracy of estimation for parasitism events and host responses (Table S1, Supporting information). Thus, to more accurately estimate latency to response, we used a bootstrap approach. Specifically, when an estimate (e.g., latency to response) was uncertain, we report the mean of 100 randomly selected (with replacement) estimates between the smallest and largest possible values. Using these estimates of latency to response, we calculated when during the laying and incubation stages hosts were most likely to respond. For these calculations, we standardized all events to clutch completion. Specifically, we subtracted the total clutch size from the clutch size at the time of parasitism and added latency to this value (i.e., responses 1 day before, on, and 1 day after clutch completion would equal −1, 0, and 1, respectively). Due to nest losses, total clutch size could not be estimated for all nests. Thus, we assumed that all warblers in this population had the mean clutch size for nonparasitized nests with known egg‐laying sequences (mean ± SE: 4.70 ± 0.05 eggs, *n* = 204). These estimates were then smoothed using kernel density estimation (using a Gaussian kernel), which resulted in the probability density functions for timing of parasitism and host response over time (Fig. 1), both including modeled uncertainty.

### Field methods to assess dynamic mimicry

Based on our preliminary observations of relatively long latency to response against some natural cuckoo eggs, we examined eggshell color and mimicry changes over this empirically derived host response period. For this investigation, conducted in 2013, we found most nests during the building stage, and these nests were checked daily until clutch completion. Each newly laid egg was numbered using a felt‐tipped pen to allow its identification. We investigated the change of eggshell coloration, across the incubation period, of warbler (*n* = 23) and cuckoo eggs (*n* = 21). Specifically, we measured the last laid warbler egg on the day of laying, each cuckoo egg as soon as they were detected (mean ± SE: 1.39 ± 0.01 days after laying), and all eggs again at 4 and 8 days after laying. The total incubation length for the cuckoos and the warbler is 11–14 days (Wyllie 1981). Three cuckoo eggs and one warbler egg disappeared prior to the measurement on the eighth day, and we used all available data for our analyses (see below). Cuckoo eggs were generally detected by the morning after they were laid, because cuckoos typically lay in the afternoon (Wyllie 1981). All eggs came from separate nests (except of two cuckoo eggs laid by different females into one nest). We left all warbler eggs in their nests throughout the incubation period; however, a subset of the cuckoo eggs (*n* = 10 of the total sample of 21 eggs) were transferred to an incubator (HEKA‐kongo; HEKA‐brutgeräte, Rietberg, Germany) within 1 h of the first color measurement. This assured that at least some (*n* = 10) cuckoo eggs would remain available for our research throughout the incubation period (i.e., not be ejected from the nest). We found no reflectance or perceived chromatic or achromatic differences between naturally and artificially incubated cuckoo eggs (Appendix S1, Supporting information), and therefore, all cuckoo eggs were combined for all analyses (Table S2, Supporting information).

An observer (M.Š.) measured the spectral reflectance of eggshells across the avian visible range (300–700 nm) using a portable spectrometer (Jaz Spectrometer, Ocean Optics, Dunedin, FL) with a built‐in pulsed xenon light source (Jaz‐PX Lamp Module, Ocean Optics Dunedin, FL). Measurements were taken at a 45° angle relative to the eggshell surface to reduce specular glare (Montgomerie 2006). Each measurement covered ~1 mm^2^ area of the eggshell surface. These color measurements were performed under standardized conditions within a dark box, specifically designed for this purpose, and were relative to a white standard (WS‐1; Ocean Optics). We took nine measurements, three from each of three eggshell regions (blunt pole, middle part, and sharp pole) and used the mean of these nine reflectance spectra for each egg at each time period (initial, after 4 days, and after 8 days). We used markings (using a felt‐tipped pen) to assure we always measured the same areas of the eggshells. To eliminate measurement errors due to marked curvature of eggshell surface, we avoided taking measurements from the extremes of the egg poles (Fecheyr‐Lippens et al. 2015) and also avoided large dark spots because their very low reflectance could bias mean eggshell reflectance, and because mimicry has been established in this host–parasite system based on ground color measurements (Igic et al. 2012).

### Perceptual color analysis

We used the software package “pavo” in the R statistical environment (Maia et al. 2013), for all color processing. First, eggshell reflectance spectra were summarized every nanometer and smoothed using a locally weighted Gaussian second‐degree polynomial regression with a smoothing span on 0.25 nm. Visual inspection of each spectrum for anomalous readings (e.g., flat spectra or noise) found no evidence of erroneous measurements. For each egg, we calculated quantum catch for each photoreceptor using the photoreceptor sensitivity and density estimates for the *Cyanistes caeruleus* Linnaeus (blue tit) published in TetraColourSpace (Stoddard and Prum 2008), based on solar irradiance measurements taken at open nests (Avilés et al. 2008). We chose the blue tit as an established model species for approximating the great reed warbler's vision (following, Avilés 2008; Honza et al. 2011, 2014; Stoddard and Stevens 2011; Drobniak et al. 2014; Šulc et al. 2016); however, it is important to note that visual abilities of differ among species (Hart and Vorobyev 2005) and these models are intended only to approximate host perception. Using this visual system information, we then modeled perceivable chromatic and achromatic differences between eggs using receptor noise‐limited visual models accounting for neural noise (Vorobyev and Osorio 1998; Vorobyev et al. 1998; Siddiqi et al. 2004). This approach produces an estimate of discriminability that is expressed in units of just noticeable differences (JND), for chromatic and achromatic aspects of coloration, where values above one would be noticeable under ideal viewing conditions, but not under suboptimal conditions (Siddiqi et al. 2004). Here, we use these estimates to place the magnitude of these color changes into an ecologically relevant context. We assume that warblers make comparisons in real time (e.g., between eggs viewed on day 4) rather than across time periods (e.g., comparing a particular egg on day 1 to that same egg on day 8) which would impose substantial memory costs (Dukas 1999) and elevated risks of rejection errors (Antonov et al. 2008; Samas et al. 2014).

### Statistical analyses

We used linear mixed‐effect models to determine whether the coloration of individual eggs changed over time. Thus, we focused on the differences between day 4 and initial measurements, and the differences between day 8 and initial measurements. We did so separately for three response variables. First, we assessed the physical changes in reflectance spectra over the incubation period by examining differences in mean brightness (hereafter reflectance differences), which may not necessarily be fully perceivable to hosts. Second, we examined the just noticeable chromatic differences in eggshell coloration (hereafter, chromatic differences). Third, we examined the just noticeable achromatic differences in eggshell coloration (hereafter achromatic differences). These models included time (categorical: after 4 or 8 days) and species (categorical: warbler or cuckoo) as predictors. Our models allowed for both random intercepts and slopes for each egg over time (as recommended by, Schielzeth and Forstmeier 2009). To aid interpretation of our model predictions, we centered and scaled chromatic and achromatic differences (Schielzeth and Forstmeier 2009; Schielzeth 2010). We square‐root‐transformed achromatic differences, prior to centering and scaling, to improve the normality of model residuals. In addition, we explored whether reflectance, chromatic, and achromatic differences changed similarly over time across species, by considering the potential interactions between time and species. We used likelihood ratio tests to compare models with and without an interaction, fitted via maximum likelihood. Including the interaction never significantly improved any of the models. We used likelihood ratio tests to evaluate the full model statistics and significance (Forstmeier and Schielzeth 2011). We used a series of model diagnostics to assess the validity of each model and identify potential outliers (following the guidelines of, Zuur et al. 2010); one warbler egg's chromatic difference value biased the perceived color change model, and therefore, this measurement was excluded to assure reliable model output.

Because the effects of eggshell color changes are not equivalent across all spectral regions (Navarro and Lahti 2014), we examined how these dynamic eggshell color shifts varied across the full avian visible spectrum using Fisher's exact *g* tests for multiple time series (Fisher 1929), which are designed to handle autocorrelated data and have been applied for other autocorrelated biological time series, such as analyzing microarray time series (Fokianos et al. 2004). In this case, these tests determined whether any wavelengths changed more than others and were based on the subtraction of each egg's initial reflectance spectrum from its reflectance spectrum measured 8 days after laying. Thus, significance would suggest that some wavelengths changed more than others, and nonsignificance would suggest that all wavelengths changed in a similar way. These analyses were run separately on each egg that was measured initially and after 8 days (*n* = 40); therefore, we applied Bonferroni corrections to these significance values and set the critical value at 0.001 (i.e., 0.05/40 trials). By employing these analyses, we avoided the arbitrary division of reflectance spectrum into subjective regions.

We used linear mixed models to predict chromatic and achromatic mimicry (i.e., the difference between warbler and cuckoo eggshell coloration) by measurement time (initial, after 4 days, after 8 days). For these tests, we compared the difference in coloration between each warbler and each cuckoo eggshell. These models allowed random intercepts and slopes for each comparison pair over time. This statistical design allowed us to determine whether the degree of mimicry changed over time. Similar to our previous analysis, chromatic differences were centered and scaled, and achromatic differences were square‐root‐transformed and then centered and scaled.

To determine whether color changes over time were significantly greater than the theoretical JND threshold of one, and thus theoretically noticeable to hosts (Honza et al. 2011, 2014; Stoddard and Stevens 2011; Drobniak et al. 2014; Šulc et al. 2016), we reran each linear mixed model fixing the intercept at zero (Eisenhauer 2003). This model specification was particularly appropriate for our analysis of color change: It has been employed for other analyses for avian color change (Hasegawa et al. 2008) and is the recommended approach to appropriately estimate group means (Schielzeth 2010). The only difference between these reanalyses and our initial models was that the dependent variables were only transformed by subtracting one JND (i.e., not centered or scaled). This has no influence on significance tests associated with other parameter estimates and was used to establish reliable estimates (and SE) for chromatic and achromatic differences. Using this approach, the *t*‐tests and associated significances for parameter estimates of measurement time do not reflect the differences between levels (Schielzeth 2010), the initial measurement, and measurements 4 and 8 days later. Instead, these parameter estimates and tests examine how each level compares to the theoretical threshold of one JND (set to zero by the transformation).

All analyses reflect marginal sums of squares and were conducted in R version 3.1.2 (R Development Core Team 2014). We present *r* ^2^ values for linear mixed models (Nakagawa and Schielzeth 2013) representing the variance explained by the fixed effects (marginal *r* ^2^, hereafter $r_{m}^{2}$) and the entire model including both the fixed and random effects (conditional *r* ^2^, hereafter $r_{c}^{2}$). All final models were recalculated via restricted maximum likelihood, and all parameter estimates and data are presented as mean ± SE.

## Results

We found that cuckoos typically laid their egg after the host laid its second egg (bootstrap estimate: 1.88 ± 0.14 host eggs, *n* = 91), which represents a substantial risk because recent evidence illustrates that these cuckoo eggs would hatch prior to the great reed warbler eggs (Geltsch et al. 2016). Hosts typically rejected these cuckoo eggs after 3.5 days (bootstrap estimate: 3.53 ± 0.30 days, *n* = 91); however, a relatively large proportion of cuckoo eggs are not rejected immediately and require relatively long periods (up to 10 days after the onset of incubation) before rejection (Fig. 2). For example, the probability that a host will reject a cuckoo egg prior to clutch completion is only ~0.46 (i.e., there is a ~0.54 probability that rejections occur after clutch completion, Fig. 2), and our data indicate that even 5 days after clutch completion (~8 days exposure to a cuckoo egg), there is a ~0.12 probability (Fig. 2) that the warbler will still reject the cuckoo egg, suggesting that the host continues to evaluate eggs in the nest well after incubation has begun.

**Figure 3:**
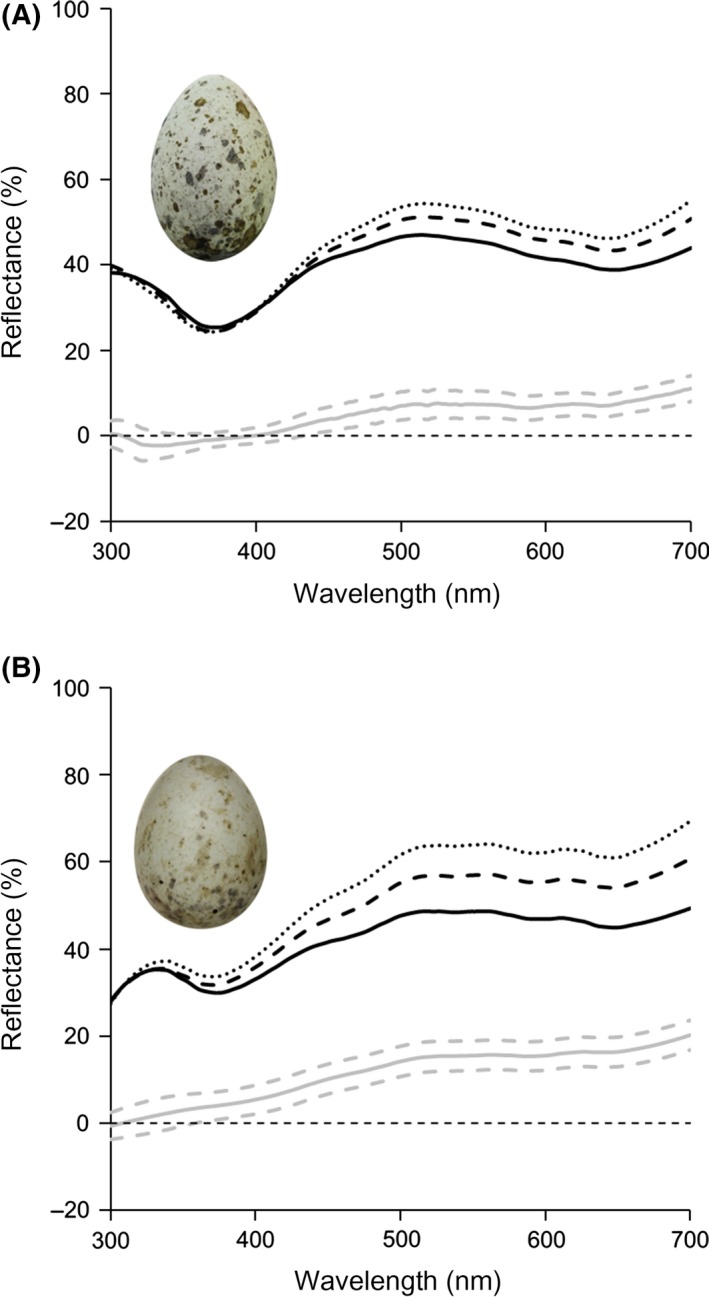
Reflectance spectra from (A) great reed warbler and (B) common cuckoo eggs measured at the time of laying (solid lines), 4 days after laying (dashed lines), and 8 days after laying (dotted lines). Each species’ average change in eggshell coloration (day 8 reflectance minus initial reflectance; solid gray) is illustrated along with the (gray dashed lines) 95% family‐wise confidence intervals. A straight thin dashed line is placed at zero percent reflectance to differentiate positive and negative changes in reflectance over time.

**Figure 4:**
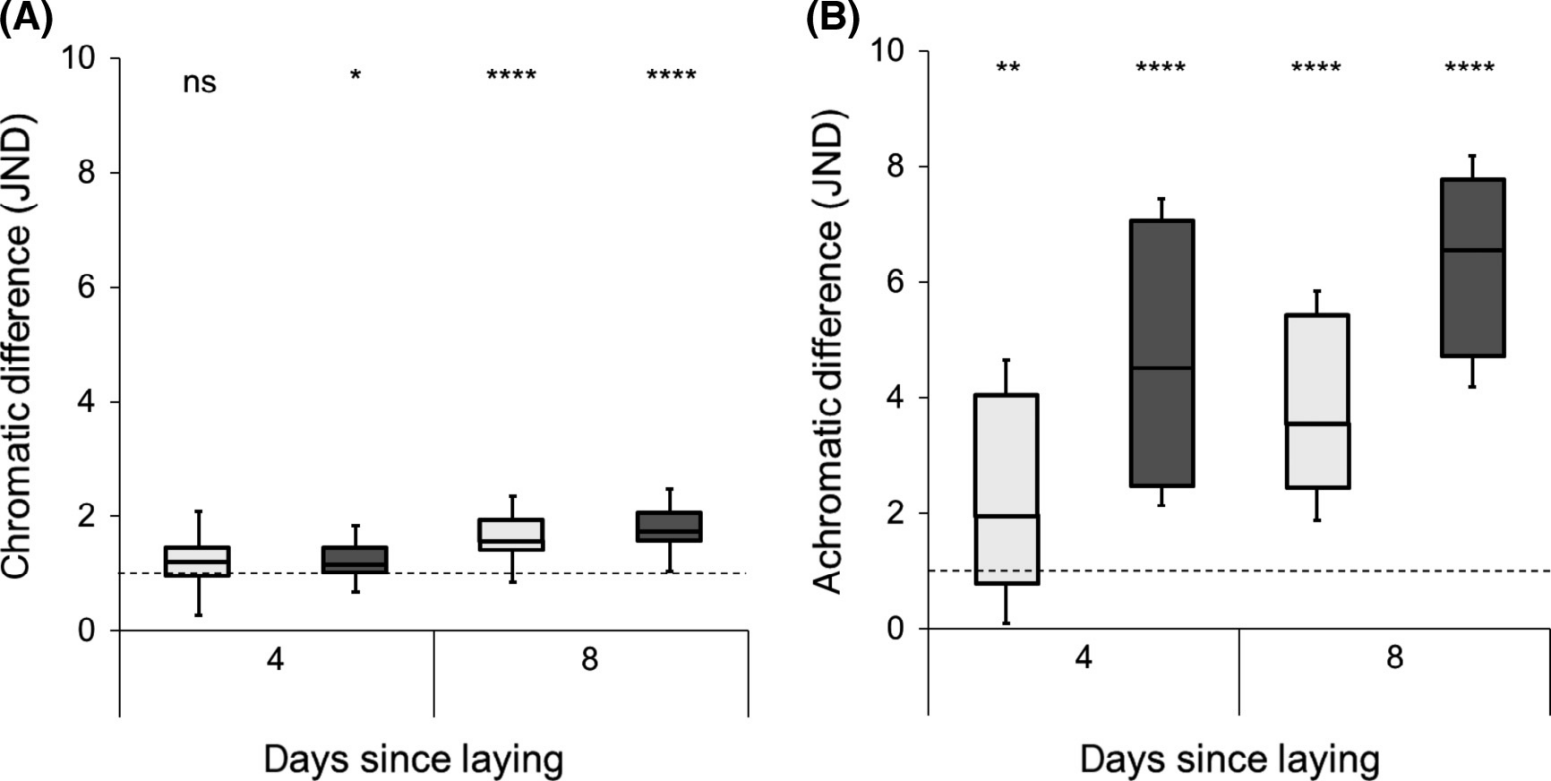
Boxplots illustrating the predicted chromatic (A) and achromatic (B) differences (JND units) from linear mixed random slope and intercept models for warblers (light gray) and cuckoos (dark gray). Whiskers represent the minimum and maximum. Significances indicate simple main effects that are >1 JND (dashed line, ns >0.05, *≤0.05, **≤0.01, ****≤0.0001). For the significance between species, please see “Results”. JND, just noticeable differences.

Warbler and cuckoo eggshells brightened over time, with greater changes in cuckoo eggshell reflectance (whole model: $r_{m}^{2}$ = 0.16, $r_{c}^{2}$ = 0.87, $\chi_{1}^{2}$ = 15.07, *P < *0.0001; time: *β* = 0.44 ± 0.16, *t*_39_ = 2.81, *P* = 0.008; species: *β* = 0.71 ± 0.24, *t*_42_ = 2.99, *P* = 0.005). These changes in eggshell reflectance were biased toward longer wavelength ranges (Fisher's exact g, all *P* values < 0.001; Fig. 3). Our visual models showed that these changes in coloration resulted in avian perceivable chromatic and achromatic differences for host and parasite eggshell coloration across the incubation period (chromatic whole model: $r_{m}^{2}$ = 0.16, $r_{c}^{2}$ = 0.88, $\chi_{1}^{2}$ = 24.20, *P < *0.0001; time: *β* = 0.71 ± 0.13, *t*_38_ = 5.65, *P < *0.0001; species: *β* = 0.13 ± 0.22, *t*_42_ = 0.60, *P* = 0.55; achromatic whole model: $r_{m}^{2}$ = 0.21, $r_{c}^{2}$ = 0.85, $\chi_{1}^{2}$ = 19.95, *P < *0.0001; time: *β* = 0.50 ± 0.18, *t*_39_ = 2.81, *P* = 0.008; species: *β* = 0.79 ± 0.21, *t*_42_ = 3.28, *P < *0.001; Fig. 4). Specifically, we found that host and parasite eggshell colors had similar chromatic JND (see the effect of species above), such that only by day 8 the magnitude of change should be perceivable to hosts (Fig. 4). By contrast, temporal changes differed between host and parasitic eggs, and our models illustrate that hosts should be able to perceive achromatic differences from the initial measurement at both 4 and 8 days after laying (Fig. 4).

**Figure 5:**
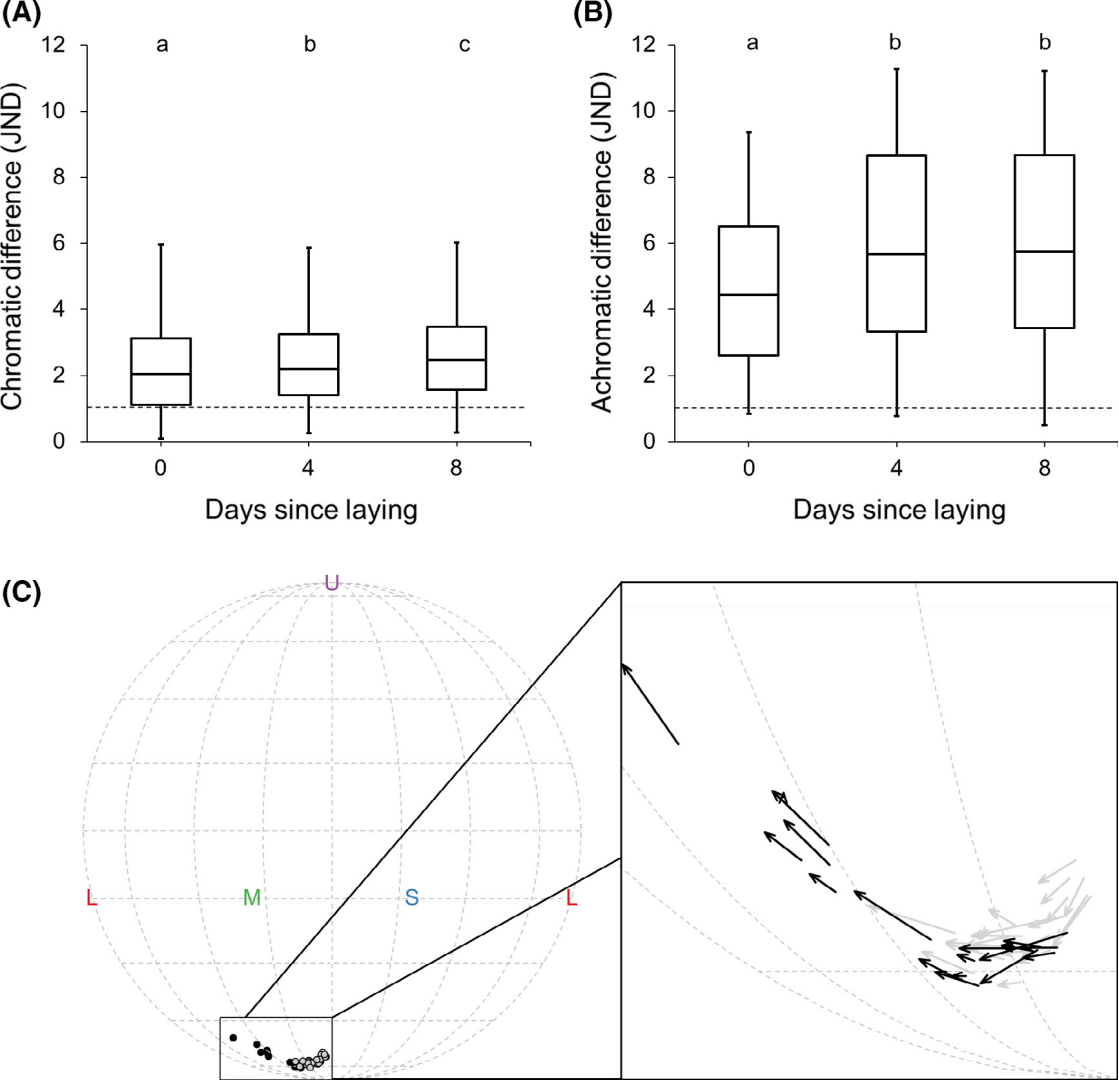
Boxplots illustrating the predicted chromatic (A) and achromatic (B) differences between all warbler and all cuckoo eggs. Whiskers represent the minimum and maximum. All differences were significantly >1 JND (dashed line, all *P* < 0.0001). Letters above bars represent significant differences in JNDs when taken at different measurement times. In addition, we illustrate (C) the hue distribution of warbler (open circles) and cuckoo (black circles) eggshell colors in a Mollweide projection, with letters representing the ultraviolet (U), short (S), medium (M), and long (L) wave‐sensitive photoreceptors. In the inset, arrows illustrate the trajectory of individual eggshell's color change from the time of laying to 8 days after laying for warblers (gray) and cuckoos (black). JND, just noticeable differences.

Our models show that cuckoo eggshell coloration, both chromatic and achromatic, was always noticeably different (i.e., >1 JND) from warbler eggshell coloration (Fig. 5A and B). Over the incubation period, cuckoo eggshell mimicry became significantly worse, in terms of both chromatic differences (whole model: $r_{m}^{2}$ = 0.009, $r_{c}^{2}$ = 0.94, $\chi_{1}^{2}$ = 59.52, *P* < 0.0001; after 4 days: β = 0.13 ± 0.03, *t*_877_ = 5.18, *P* < 0.0001; after 8 days: *β* = 0.23 ± 0.03, *t*_877_ = 7.97, *P* < 0.0001) and achromatic differences (whole model: $r_{m}^{2}$ = 0.02, $r_{c}^{2}$ = 0.84, $\chi_{1}^{2}$ = 51.57, *P* < 0.0001; after 4 days: *β* = 0.31 ± 0.05, *t*_877_ = 6.33, *P* < 0.0001; after 8 days: *β* = 0.31 ± 0.05, *t*_877_ = 5.79, *P < *0.0001). Initially, cuckoo eggshell colors were only slightly browner than host eggshell colors, but this chromatic mismatch was exacerbated over time (Fig. 5C).

## Discussion

Eggshell colors can change over time (Cassey et al. 2012; Hanley et al. 2013b; Navarro and Lahti 2014), and our work demonstrates that eggshell color mimicry should be considered a dynamic rather than a static trait during the laying and incubation periods. Specifically, the eggshell coloration of the great reed warbler host and its parasite, the common cuckoo, brightened over incubation. We also demonstrate that these changes may have functional consequences, as egg rejection extends into the incubation period (Fig. 2) just like in the majority of brood parasite hosts tested so far (e.g., Davies and Brooke 1989; Antonov et al. 2008; Grim et al. 2011, 2014; Samas et al. 2014). We found that these temporal color changes did not occur evenly across the avian visual spectrum. Changes in eggshell spectral reflectance occurred mainly at longer wavelengths (Fig. 3), which led to hue and chromatic shifts from eggshell colors that were more blue‐green to those that were more brown (Fig. 5C). This also explains why various proportion‐based eggshell color metrics, such as ultraviolet and blue‐green chroma, have been found to change between subsequent measurements (Cassey et al. 2010; Moreno et al. 2011; Navarro and Lahti 2014), and why these metrics change at different rates (Cassey et al. 2010; Hanley et al. 2013a). Importantly, our findings illustrate that the changes in eggshell coloration of this magnitude should be noticeable to hosts.

Although the eggs of both species brightened, we found cuckoo eggshell coloration brightened more than warbler eggshell colors did and, based on our visual models, differences of this magnitude would be perceivable to the host. In this population, hosts and parasites share similar eggshell pigment chemistry (Igic et al. 2012), which most likely explains why the eggshell coloration changed in the similar way (e.g., greater brightening of long wavelengths) for both species. However, the exact mechanism behind eggshell color change, and why there were greater color changes at longer wavelengths (i.e., green and red) relative to shorter wavelengths (i.e., ultraviolet and blue), has yet to be determined. Moreno et al. (2011) explained eggshell color change as chemical degradation of eggshell pigments, but a number of other mechanisms are plausible (Yousif and Haddad 2013). For example, both eggshell pigments are known to photodegrade (Lightner and Crandall 1972; Ericson et al. 2003), particularly in the presence of oxygen (Ericson et al. 2003). Alternatively, the eggshell color changes that we detected may be the result of oxidative degradation or thermal oxidative degradation (Feldman 2002).

Variation in temporal color changes between species may be an unavoidable by‐product of subtle variation in eggshell pigment chemistry or structure (Hanley et al. 2015a; Igic et al. 2015a), and while color changes due to soiling are possible (Mayani‐Parás et al. 2015) we found no evidence to support this form of color change (Appendix S1, supporting information). Both eggshell pigments fade at slightly different rates (Lightner and Crandall 1972; Wojaczyński 2014), and thus, future research should examine how the absolute and relative concentrations eggshell pigments result in varying rates of color change. In addition, a number of natural materials have been found to stabilize protoporphyrin from degradation (Crowley 1999), with ultraviolet absorbers being an important class of polymer stabilizer (Yousif and Haddad 2013). Recent research has found that the eggshell cuticle absorbs ultraviolet light (Fecheyr‐Lippens et al. 2015), suggesting that the cuticle may function as a natural photostabilizer for eggshell pigmentation. We encourage future field experimentation to explore the role of eggshell chemistry and structure on the eggshell color degradation process. However, whatever the mechanism, we cannot exclude the possibility that the rates of change are under selection.

In this study, we found that despite similar eggshell color changes, the degree of chromatic and achromatic eggshell mimicry between warbler and cuckoo eggs changed dynamically over the incubation period. Specifically, the chromatic match between cuckoo and warbler eggshell coloration progressively worsened from laying to shortly before hatching. Achromatic mimicry also worsened but was poorest 4 days after laying. This may be explained by differences in the magnitude of brightening between the two species over time (Fig. 3), which were biased toward long wavelengths. Thus, while within‐egg chromatic differences were statistically similar between species, the chromatic differences between species increased, most likely because these biased changes in brightness resulted in relatively larger changes in hue and chroma for cuckoos than warblers (Fig. 5).

This dynamic mimicry suggests an under‐appreciated aspect of coevolutionary arms races. Rapid color changes may be beneficial for the great reed warblers, which generally discriminate mimetic cuckoo eggs within 1–2 days (Moskát et al. 2014a; Trnka and Grim 2014), although we show that they continue to reject well into the incubation period (up to 10 days; Fig. 2). Some warblers may delay decision making to exploit temporal shifts in mimicry, or those eggs may go unnoticed until their colors change, and this could explain why rejections occur either relatively quickly (e.g., ~90% of rejections occur within 1–2 days) or relatively late (~10% of rejections occur within 4–6 days; data from, Trnka and Grim 2014). Thus, cuckoos may optimally invest in eggshell chemistry to sustain eggshell color mimicry during their hosts’ decision‐making period (Moskát and Hauber 2007; Antonov et al. 2008). Reliable temporal egg color degradation can provide valuable information on the timing of egg laying (Hanley et al. 2013b) and has been found to be the salient recognition cue for egg rejection in the *Crotophaga major* Gmelin (greater ani), which is a conspecific avian brood parasite (Riehl 2010).

Additionally, our findings raise concerns about the accuracy of eggshell color comparisons and estimates of eggshell color mimicry for studies that have not considered egg age in either the experimental design, or the statistical analysis. It is possible that these uncontrolled temporal components of avian eggshell coloration may explain why support for some hypotheses has been so mixed (Reynolds et al. 2009; Cherry and Gosler 2010). Moreover, our findings suggest that if eggshell color measurements and behavioral responses to those eggs are mismatched (e.g., spectral reflectance measured on fresh eggs and then partially incubated eggs are used later in experimental trials), experimental estimates of behaviors related to those egg colors would be either under‐ or overestimated (Hauber et al. 2015). The magnitude of eggshell color degradation that we detected can result in moderate increases in ejection probabilities in warblers (Hauber et al. 2015). Moreover, this degree of color difference (~2 JND; Fig. 4) is close to the scale of mimicry achieved by the cuckoo (Stoddard and Stevens 2011), where there is approximately a 3 JND difference in eggshell color mimicry between the least and most mimetic gentes: *Prunella modularis* Linnaeus (the dunnock; ~4.5 JND) and *Lanius collurio* Linnaeus (the red‐backed shrike; ~1.5 JND), respectively.

Although our research provides insight into the process and consequences of eggshell color degradation, there is currently no method to correct for these temporal color shifts. Therefore, we recommend that researchers comparing eggshell coloration, especially field researchers, consider egg age. Further in situ research is required to determine the mechanism behind the dynamic eggshell color change, because the degradation of pigment‐based colors is influenced by exposure to light, air, and the pigment's substrate (Gervais et al. 2014), which varies across the incubation period (Yu et al. 2016). We encourage researchers to investigate and employ specific methods to correct for the effects of eggshell color changes. This would allow for greater flexibility and comparability between studies. These findings of eggshell color changes are not only important for statistical analysis and study design, but also biologically relevant because they alter eggshell color mimicry over time. Our findings illustrate that these eggshell color changes would most likely be perceptually noticeable to hosts, and therefore, they may provide the basis for the study of unexplored modes of communication and the functions of avian eggshell coloration.

Across the full phylogenetic diversity of birds, both eggshell pigments (Hanley et al. 2015a) and eggshell color changes appear ubiquitous (for diverse taxa see, Cassey et al. 2010; for diverse taxa see, Moreno et al. 2011; Hanley et al. 2013a, 2013b; Navarro and Lahti 2014). Therefore, our finding of dynamic eggshell color mimicry may be a widespread phenomenon across all heterospecific (Stoddard and Stevens 2010) and conspecific (Samas et al. 2014) brood parasites. The temporal changes in eggshell mimicry that we document may aid host egg discrimination, and therefore, these eggshell color changes may have important coevolutionary consequences.

## Data Accessibility

The supporting information associated with this manuscript contains all data used.

## Conflict of Interest

None declared.

## Supporting information


**Table S1.** Behavioral data.

**Table S2.** Eggshell reflectance.

**Appendix S1.** Extended materials and methods.
