# Supplementary material for: Dynamic egg color mimicry
Source: Ecol Evol. 2016 May 24;6(12):4192–202. doi: 10.1002/ece3.2187 (PMC4972242; doi:10.1002/ece3.2187)
Supplement: Supplementary file 3 — Appendix S1. Extended materials and methods. [file ECE3-6-4192-s003.docx]

**Supporting Information** accompanying the manuscript:

Daniel Hanley, Michal Šulc, Patricia L. R. Brennan, Mark E. Hauber, Tomáš Grim, and Marcel Honza (2016) Dynamic egg colour mimicry. Ecology and Evolution. xx: xx

**Extended materials and methods**

(*a*) *Artificial incubation*

Great reed warblers are skilled at rejecting cuckoo eggs. Thus to assure that we had eggshell reflectance measurements across the entire incubation period, we removed a subset of cuckoo eggs (N = 10 of 21) and placed them in an incubator (HEKA-kongo, HEKA-brutgeräte), with temperature and relative humidity ranging from 37.3-37.5 °C and 63-67%, respectively. This incubator was exposed to both natural light, through a window, as well as periodic artificial light from an interior light source.

(*b*) *Statistical analyses*

To determine if the coloration of individual eggs changed over time as a result of transferring eggs into the incubator, we used linear mixed effect models to predict differences in mean brightness (hereafter reflectance differences), chromatic, or achromatic coloration of each cuckoo egg by measurement time (categorical: after 4 or 8 days; reference level: 4 days), and the form of incubation (categorical: artificial or natural; reference level: artificial incubation). Our models allowed for both random intercepts and slopes for each egg over time (as recommended by, Schielzeth & Forstmeier 2009). All dependent variables were centred and scaled (Schielzeth & Forstmeier 2009; Schielzeth 2010) and achromatic differences were square root transformed prior to centring and scaling to improve the normality of model residuals. We examined potential interactions between time and form of incubation using likelihood ratio tests comparing models with and without an interaction, fit via maximum likelihood. Interactions did not significantly improve any of the models. We used likelihood ratio tests to evaluate the full model statistics and significance (Forstmeier & Schielzeth 2011). We used a series of model diagnostics to assess the validity of each model and identify potential outliers (following the guidelines of, Zuur, Ieno & Elphick 2010). The reported *r*^2^ values are designed for linear mixed models (Nakagawa & Schielzeth 2013) that represent the variance explained by the fixed effects (marginal *r*^2^, hereafter *r*^2^_m_) and the entire model including both the fixed and random effects (conditional *r*^2^, hereafter *r*^2^_c_). To assure reliable estimates we refit all models via restricted maximum likelihood and report these estimates. All analyses were conducted in R version 3.1.2 (R Development Core Team 2014) and parameter estimates are presented as mean ± SE.

**Supplemental Results**

Naturally and artificially incubated cuckoos showed similar changes in reflectance (*whole model*: r^2^_m_ = 0.10, r^2^_c_ = 0.88, χ^2^_1_ = 7.40, *p* = 0.02; *time*: β=0.58±0.21, t_17_ = 2.73, *p* = 0.01; *form of incubation*: β = 0.23±0.35, t_19_ = 0.65, *p* = 0.52), chromatic (*whole model*: r^2^_m_ = 0.29, r^2^_c_ = 0.90, χ^2^_1_ = 16.31, *p* < 0.0001; *time*: β=0.85±0.20, t_17_ = 4.31, *p* < 0.001; *form of incubation*: β = 0.66±0.32, t_19_ = 2.02, *p* = 0.06), and achromatic differences (*whole model*: r^2^_m_ = 0.08, r^2^_c_ = 0.84, χ^2^_1_ = 3.17, *p* = 0.20; *time*: β=0.52±0.29, t_17_ = 1.79, *p* = 0.09; *form of incubation*: β = −0.01±0.24, t_19_ = −0.05, *p* = 0.96); therefore, we pooled naturally and artificially incubated cuckoo eggs for main analyses (see Material and methods and Results in the main text).

**Supplemental References**

Forstmeier, W. & Schielzeth, H. (2011) Cryptic multiple hypotheses testing in linear models: overestimated effect sizes and the winner’s curse. *Behavioral Ecology and Sociobiology*, **65**, 47–55.

Grim, T. (2006) The evolution of nestling discrimination by hosts of parasitic birds: why is rejection so rare? *Evolutionary Ecology Research*, **8**, 785–802.

Mason, P. & Rothstein, S.I. (1986) Coevolution and avian brood parasitism: cowbird eggs show evolutionary response to host discrimination. *Evolution*, **40**, 1207–1214.

Nakagawa, S. & Schielzeth, H. (2013) A general and simple method for obtaining R^2^ from generalized linear mixed-effects models (ed RB O’Hara). *Methods in Ecology and Evolution*, **4**, 133–142.

Navarro, J.Y. & Lahti, D.C. (2014) Light dulls and darkens bird eggs. *PloS one*, **9**, e116112.

R Development Core Team. (2014) R: a language and environment for statistical computing.

Schielzeth, H. (2010) Simple means to improve the interpretability of regression coefficients. *Methods in Ecology and Evolution*, **1**, 103–113.

Schielzeth, H. & Forstmeier, W. (2009) Conclusions beyond support: overconfident estimates in mixed models. *Behavioral Ecology*, **20**, 416–420.

Zuur, A.F., Ieno, E.N. & Elphick, C.S. (2010) A protocol for data exploration to avoid common statistical problems. *Methods in Ecology and Evolution*, **1**, 3–14.
